# Dietary Phytosterols Protective Against Peptic Ulceration

**DOI:** 10.4021/gr328w

**Published:** 2011-07-20

**Authors:** Frank I Tovey, Doga Capanoglu, G. John Langley, Julie M Herniman, Serhat Bor, Omer Ozutemiz, Michael Hobsley, Karna Dev Bardhan, Bruno Linclau

**Affiliations:** a Division of Surgery and Interventional Science, University College, London, UK; b Department of Gastroenterology, Ege University, Bornova, Turkey; c School of Chemistry, University of Southampton, UK; d Department of Gastroenterology, Rotherham General Hospital, UK

**Keywords:** Phytosterols, Gastroprotection, Duodenal ulceration, NSAIDs

## Abstract

**Background:**

In developing countries the prevalence of duodenal ulceration is related to the staple diet and not to the prevalence of Helicobacter pylori. Experiments using animal peptic ulcer models show that the lipid fraction in foods from the staple diets of low prevalence areas gives protection against ulceration, including ulceration due to non-steroidal anti-inflammatory drugs (NSAIDs), and also promotes healing of ulceration. The lipid from the pulse Dolichos biflorus (Horse gram) was highly active and used for further investigations. Further experiments showed the phospholipids, sterol esters and sterols present in Horse gram lipid were gastroprotective. Dietary phospholipids are known to be protective, but the nature of protective sterols in staple diets is not known. The present research investigates the nature of the protective phytosterols.

**Methods:**

Sterol fractions were extracted from the lipid in Dolichos biflorus and tested for gastroprotection using the rat ethanol model. The fractions showing protective activity were isolated and identification of the components was investigated by Gas Chromatography-Mass Spectrometry (GC-MS).

**Results:**

The protective phytosterol fraction was shown to consist of stigmasterol, β-sitosterol and a third as yet unidentified sterol, isomeric with β-sitosterol.

**Conclusions:**

Dietary changes, affecting the intake of protective phospholipids and phytosterols, may reduce the prevalence of duodenal ulceration in areas of high prevalence and may reduce the incidence of recurrent duodenal ulceration after healing and elimination of Helicobacter pylori infection. A combination of phospholipids and phytosterols, such as found in the lipid fraction of ulceroprotecive foods, may be of value in giving protection against the ulcerogenic effect of NSAIDs.

## Introduction

There is strong evidence, especially from India, China, Africa and Malaysia, that the prevalence of duodenal ulceration in different geographical regions is related to the traditional staple diet of the area [1-9] and has no relationship to the prevalence of Helicobacter pylori infection [10-12]. A higher prevalence is found in areas where the staple diet is principally milled rice, refined wheat or maize, yams, cassava, sweet potato or green bananas, and a lower prevalence in areas where the staple diet is based on unrefined wheat or maize, soya, certain millets or certain pulses. These diets and individual foods have been investigated in several rat peptic ulcer models, and the results confirmed the ulceroprotective activity of the foods predominating in the diet in the lower duodenal ulcer prevalence areas. The experiments showed that the protective activity lay in the lipid component of these foods [13]. The pulse Dolichos biflorus or ‘Horse gram’ and its lipid fraction showed greatest protection, and this was used for further investigations using rat peptic ulcer models. Three rat models producing gastric ulceration were used to show protective activity. These included pyloric ligation, a chronic long term model that involved 2 weeks pre-feeding with the test substance, and two acute models using ethanol or aspirin, that employed a single dose of the test substance. In addition, a further acute model used cysteamine to produce duodenal ulceration. The lipid gave protection in all the above models when given topically into the stomach. In addition, it was protective when give intramuscularly in the pyloric ligation, cysteamine and alcohol models [14].

The lipid fraction from Horse gram was also shown to promote healing of gastric ulceration produced by topical application of acetic acid to the surface of the stomach, and duodenal ulceration produced by cysteamine. It also protected mast cells from degranulation in response to ulcerogenic stored rice bran oil instilled into the stomach of rats 6 hours after pyloric ligation [14]. Stored rice bran oil becomes rancid, developing ketoaldehydes which are ulcerogenic. Fresh rice bran oil is protective [9].

Having confirmed the protective activity of the lipid fraction from Horse gram, different fractions of the lipid were tested for protective activity. The phospholipid, sterol ester and sterol fractions were shown to be protective [14].

The phospholipid fraction was protective in both the acute and chronic models. Our earlier experiments using the rat ethanol model on phospholipids obtained from Sigma showed that, whereas phosphatidyl choline was not protective, phosphatidyl ethanolamine, dipalmitoyl phosphatidyl ethanolamine, dipalmitoyl phosphatidyl choline, and di-gamma-linoleic acid phosphatidyl choline all gave significant protection [15] (Dipalmitoyl phosphatidyl choline is not found in plant material).

Sterols were protective in the acute models, but not in the pyloric ligation chronic model. Sterol esters were protective only in the chronic model. This could be explained by the fact that sterols are poorly absorbed in the small intestine so that their action on the gastroduodenal mucosa in the acute model is topical, whereas sterol esters are absorbed and therefore could have a systemic effect in the chronic model [15].

There is very little information about the possible ulceroprotective effect of sterols and related substances. We have found only four reports about the ulceroprotective activity of sitosterols in animal models [16-19]. Two of these compare the protective activity of β-sitosterol with a glycoside. One is in Chinese [18] and reports on the antigastroulcerative activity of β-sitosterol-glycoside and its aglycone in rats using acetic acid and cold stress models, the effect being greater with the aglycone. The other paper reports gastroprotective activity of β-sitosterol and sitosterol-3-0-β-glucoside in rat ethanol, aspirin, histamine, pyloric ligation and 8 day glucose diet models [19], while the sitosterol glycoside in this report being more effective than sitosterol. Other papers report on the protective activity of sterolins (sterol glycosides). Two such papers report [20, 21] that unripe green banana (Musa Paradisiaca) in the Gangetic basin is protective against experimental ulceration in rats, using the aspirin model, between August and October when it contains a combination of four sitosterolins (sitoindoside I and II, sitosterol gentobioside and sitosterol myo-inosityl-β-D-glucoside). A further paper, using mice restraint models, reports the ulceroprotective activity of a mixture of steryl-β-D-glucosides [22].

There have been several reports about the gastroprotective effects of phospholipids [23-26]. They are integral components of cell membranes and of gastric and duodenal mucosal cells. They contribute to the viscosity of the surface layer of mucus [27], and prevent intracellular acidification. The acyl side chains of unsaturated phospholipids supply unsaturated fatty acids for the synthesis of prostaglandins. Lecithins and to a lesser extent cephalins are reported to have a surfactant effect which protects against experimental gastric ulceration [28-30]. This effect, particularly that of dipalmitoyl phosphatidyl choline (DPPC), is enhanced in the presence of neutral lipids allowing the formation of a micro-emulsion [30]. Thus phospholipids can have both a systemic and a topical effect in mucosal protection. It has been shown in humans that DPPC or phosphatidyl choline protects against the injurious effects of NSAIDs [32-35].

As reported earlier [14], the crude Horse gram lipid was protective both when given topically and parenterally in the pyloric ligation, cysteamine and alcohol models. Phospholipids when given intramuscularly are absorbed locally and do not have a systemic effect. This means that the parenteral activity of Horse gram lipid must lie in the sterol/sterol ester fraction present in the lipid.

In our earlier investigations into the ulceroprotective activity of sterols, a sterol preparation from Horse gram lipid was obtained by thin layer chromatography (TLC). The band that corresponded with 60% pure β-sitosterol obtained from soya (Sigma) was shown to be protective. This fraction showed protective activity in the rat ethanol model in doses down to 130 µg [15]. Analysis by analytical High Performance Liquid Chromatography (HPLC) consistently showed that, between different samples obtained via repeated extractions, the major component consisted of β-sitosterol. Other sterols, such as dihydrobrassicasterol, stigmasterol and campesterol were shown to be present in various amounts, plus an unidentified sterol. The unidentified sterol was thought to be Δ-7 stigmastenol (Schottenol), but no suitable reference compound was available for its identification despite extensive searching.

The late Dr. A. Paul Jayaraj conducted all the extractions and bioassays in the earlier stages of this research. The object of the present research was to identify the individual sterols present in the sterol fraction that are responsible for giving the protection. It is the outcome of collaboration between the School of Chemistry, Southampton University, the Department of Gastroenterology at the Ege University in Bornova, Turkey and the Department of Surgery, University College, London.

## Methodology

The procedure fell into three parts: (1) preparation of the sterol fraction present in Horse gram corresponding to 60% β-sitosterol and separation of the fraction into the component sterols; (2) bioassays using the rat ethanol model to determine the ulceroprotective activity of the sterol fractions; (3) characterisation of the active sterol fractions by Gas Chromatography Mass Spectrometry and Electron Ionisation.

### Preparation of sterol fractions

#### Preparation of a whole sterol extract from Horse gram and isolation of the component fraction corresponding to 60% β-sitosterol by preparative column chromatography and bioassay

The Horse gram was powdered and the polar fractions removed by extraction with 70% ethanol and 5% acetic acid, and after filtration by further treatment with 66% ethanol in water. The residue was then treated with hexane for 3 hours at 50°C using a reflux condenser. The hexane was evaporated leaving a viscous green fluid extract. Two hundred gram of horse gram yielded 1 - 1.4 g of crude extract. Isolation of the sterol fraction was achieved using preparative column chromatography (silica gel) using hexane/acetone 80:20 to 75:25 as eluent, where the fraction corresponding to a β-sitosterol standard [60% pure β-sitosterol (Sigma)] was isolated. The resulting fraction was tested on the ethanol model and the presence of protective activity was confirmed (see results below).

#### Separation of the sterol fraction corresponding to β-sitosterol into component sterol fractions by preparative HPLC and bioassays

A further preparation of the crude sterol extract (5.98 g) was made as above. Purification was effected by gradient column chromatography (silica gel) using hexane/acetone 80:20 to 75:25 as eluent. The fractions (20 mL) were analysed by TLC (hexane/Et_2_O 60:40) against the β-sitosterol reference sample. Two fractions, F19 and F20, which contained compound(s) corresponding to the β-sitosterol band were selected. These two fractions were each further purified by preparative HPLC (normal phase silica gel). This resulted in fractions F23, F24, F32, F33 and F34 which were selected for bioassay in Turkey (600 g of Horse gram yielded 0.06 g of F23 and 0.023 g of F24). Fractions 23/24 showed highly significant protective activity (see results below) and were selected for identification of the component sterols.

### Bioassays: rat ethanol model

Female Wistar rats weighing 200 g were kept in individual narrow wide meshed cages to prevent coprophagy and fasted with regards to solid food for 18 hours. They were deprived of water for the last 4 of the 18 hours. The test substance was given in 0.1 mL of vehicle, sunflower oil, into the stomach by orogastric tube. Thirty minutes later, 1 mL of 80% ethanol was given intragastrically. The rats were sacrificed 1 hour later. Their stomachs were opened along the greater curve and any stomachs containing feces were discarded. The stomachs were pinned out on a board for examination. Ulceration was confined to the corpus and was linear. The total length of ulceration in each animal was measured in millimetres with a planimeter.

Controls were done throughout using 0.1 mL of the vehicle.

### Characterisation

#### Gas chromatography-mass spectrometry

The samples were analysed using a Thermo Trace GC-MS (Thermo Fisher Scientific, Hemel Hempstead, UK). One microlitre was injected (splitless) onto a Phenomenex Zebron ZB-5MS column (Macclesfield, UK), 30 m x 0.25 mm, 0.25 µm film thickness. The injector was heated to 220°C and helium carrier gas was set at a constant flow rate of 1.2 mL/min. The GC was held at 40°C for 4.5 minutes and then heated at a rate of 20°C/min to 320°C and held at the final temperature for 7 minutes. Mass spectrometry analysis was undertaken using electron ionisation (EI). The ionisation source was heated to 200°C, and 70 eV EI mass spectra were recorded over 20 - 500 *m/z* units at 2 scans per second. Data were recorded and analysed using Xcalibur™ 1.2 (Thermo Fisher Scientific, Hemel Hempstead, UK).

#### Electron ionisation

Direct insertion probe EI mass spectra were acquired using a normal geometry, double-focussing 70-250-SE mass spectrometer (VG Analytical, Manchester, UK). The ionisation source was heated to 200°C, and 70 eV EI mass spectra were recorded over 1000 - 20 *m/z* units at 5 seconds per decade. Data were recorded and analysed using Maspec II (Mass Spectrometry Services, UK).

### Ethical approval for rat ethanol model for bioassays

Ethical approval was given by the Animal Ethical Committee of the Faculty of Medicine, Ege University, Bornova, Turkey.

### Statistical methods

The imperative to minimise the numbers of animals used in these experiments meant that a normal distribution of the results could not be demonstrated. A non-parametric method (the Mann-Whitney U-test) was therefore used.

## Results

Results are presented in three stages.

### Stage 1: extraction and bioassay corresponding to 60% β-sitosterol

The sterol fraction as prepared in Stage 1 was tested in 4 mg doses using the ethanol model. The results are shown in Table 1 which shows that highly significant protection was obtained.

**Table 1:** Testing the Protective Properties of Whole β-sitosterol Fraction From Horse Gram (Ethanol Model, N = 10 Rats)

Controls: Sunflower oil 0.1 mL											
Rats	1		2	3	4	5	6	7	8	9	10
Ulcer length (mm)		0.62	2.46	2.64	4.92	1.01	0.64	1.63	1.47	3.18	3.16
Number of ulcers		1	3	7	13	3	4	6	5	11	9

Test: Whole sitosterol fraction 4 mg										
Rats	1	2	3	4	5	6	7	8	9	10
Ulcer length (mm)	9.39	0.00	0.23	0.33	0.65	0.20	0.33	0.50	0.00	0.40
Number of ulcers	11	0	1	2	1	1	2	2	0	2

Comparison of beta-sitosterol fraction (F) with controls (C)

Linear ulcers: Number F < C; P = 0.0065

Length: F < C; P = 0.0041

### Stage 2: further extraction and bioassays of the component sterols in the above fraction

A new batch of Horse gram was subjected to the extraction procedure as described in Stage 2.This yielded 6 major sterol fractions which were tested in batches for protective activity using the ethanol model. Only fractions F23 and F24 gave protection, using a small dose of 400 µg. Although apparently identical on TLC, they were tested separately and both gave equal highly significant protection. The bioassay was repeated for confirmation (Tables 2 and 3).

**Table 2:** Sterol Fraction 23 From Horse Gram (Series 1 and 2, Ethanol Model, N = 10 Rats)

Controls: Sunflower oil 0.1ml										
Rats	1	2	3	4	5	6	7	8	9	10
Ulcer length (mm)	3.80	4.05	3.65	3.49	3.31	3.88	3.66	3.89	2.20	Feces present
Number of ulcers	9	9	11	7	8	7	0	9	5	-

Test: Sterol fraction 23 400 mg. Series 1										
Rats	1	2	3	4	5	6	7	8	9	10
Ulcer length (mm)	3.46	0.51	0.91	1.64	1.70	1.01	1.90	3.11	0.00	0.67
Number of ulcers	6	1	2	3	4	5	6	8	0	2

Test: Sterol fraction 23 400 mg. Series 2										
Rats	1	2	3	4	5	6	7	8	9	10
Ulcer length (mm)	1.56	0.61	0.85	0.73	3.11	1.10	2.51	2.90	0.20	2.58
Number of ulcers	4	3	3	4	9	5	7	6	8	5

Comparison of beta-sitosterol fraction (F) Series 1 with controls (C)

Linear ulcers: Number F < C; P = 0.0036

Length F < C; P = 0.0006; U value = 3

Comparison of beta-sitosterol fraction (F) Series 2 with control (C)

Linear ulcers: Number F < C; P = 0.0009; U value = 4.5

Length F < C; P = 0.0008; U value = 4.0

**Table 3:** Sterol Fraction 24 From Horse Gram (Series 1 and 2, Ethanol Model, N = 10 Rats)

Controls: Sunflower oil 0.1ml										
Rats	1	2	3	4	5	6	7	8	9	10
Ulcer length (mm)	3.80	4.05	3.65	3.49	3.31	3.88	3.66	3.89	2.20	Feces present
Number of ulcers	9	9	11	7	8	7	9	9	6	-

Test: Sterol fraction 24 400 mg. Series 1 (8 rats)										
Rats	1	2	3	4	5	6	7	8	9	10
Ulcer length (mm)	1.14	1.71	0.37	1.13	0.00	0.24	0.57	1.00	-	-
Number of ulcers	2	6	2	1	0	1	2	3	-	-

Test: Sterol fraction 24 400 mg. Series 2										
Rats	1	2	3	4	5	6	7	8	9	10
Ulcer length (mm)	0.98	1.12	2.43	2.41	2.24	0.64	1.64	0.00	1.13	1.98
Number of ulcers	2	5	6	4	4	3	5	0	4	6

Comparison of beta-sitosterol fraction (F) Series 1 with controls (C)

Linear ulcers: Number F < C; P = 0.0036

Length F < C; P = 0.0006; U value = 3

Comparison of beta-sitosterol fraction (F) Series 2 with control (C)

Linear ulcers: Number F < C; P = 0.0009; U value = 4.5

Length F < C; P = 0.0008; U value = 4.0

### Stage 3: characterisation of fractions 23 and 24 by GC mass spectrometry and electron ionisation

GC-MS analysis of F23 indicated two major sterol components in the mixture, β-sitosterol and stigmasterol. The analysis of F24 showed the presence of β-sitosterol and another sterol of identical molecular weight. This indicates an isomeric sterol, for example y-sitosterol or Δ-7 stigmastenol (Schottenol). Unfortunately, despite an exhaustive search, we were unable to procure these substances as reference compounds to confirm the identity of the unknown sterol.

The findings confirmed the presence in the protective sterol fractions of β-sitosterol and of stigmasterol. A third isomer of β-sitosterol, which may be y-sitosterol or Δ7 stigmastenol (Schottenol), was present but could not be identified because of lack of reference compounds.

## Discussion

The initial findings confirmed the protective activity of the sterol fraction obtained by extraction from Horse gram. After a series of purifications, two active fractions were obtained, with β-sitosterol as the common substance. These findings support the literature reports which describe the protective activity of β-sitosterol [16-19]. However, while it cannot be excluded that β-sitosterol is the sole active ingredient, stigmasterol and the other unidentified sterol could also contribute to the activity.

The sterols present in plant material always exist in combination with sterol esters and glycosides (sterolins). Our earlier experiments showed that sterol esters were protective in the rat pyloric ligation model [15]. In the references previously quoted, there are reports of the gastroprotective activity of sterolins [18-22]. In our experiments sterolins were not tested for protective activity. They are polar and would have been removed in the method we have used for sterol extraction.

Phytosterols regulate membrane fluidity and the activity of membrane-bound enzymes [36]. They are active in reducing proton and sodium ion leaks from cell membranes [37], and are reported to be anti-inflammatory, anti-pyretic [38] and to enhance immune response [39, 40]. Experimentally, certain phytosterols present in sponges and coral, but derived from plankton, have been shown to inhibit histamine release from peritoneal mast cells in rats [41-43].

In animal peptic ulcer models, sterols (β-sitosterol and cholesterol) also potentiate the protective effect of unsaturated phospholipids [44], possibly due to enhancement of the packing of the unsaturated phospholipids in the cell membranes. They enhance the stability of phospholipid monolayers [45]. Phytosterols are reported to reduce the water permeability of phosphatidyl-choline bilayers. Sitosterol and campesterol are more efficient than cholesterol in ordering the acyl chains of lecithin bilayers. Stigmasterol is less active [46].

Our present research has shown that the sterol fraction in Horse gram is protective, when given alone, against experimental peptic ulceration. It is important to remember that, as mentioned above, the related sterolins and sterol esters which are present in Horse gram may also be protective. The phospholipid component of Horse Gram was also ulceroprotective. This means that both the sterol and the phospholipid compounds present in Horse gram and in other ulceroprotective foods may each provide protection in their own rights, but that they may also act in combination, one potentiating the other. This needs further investigation.

These findings can lead to a better understanding of the role of diet in the etiology and geographical prevalence of duodenal ulceration. Experiments on animal models [5, 8, 9, 13] showed that the lipid fraction present in wheat or fresh rice bran, unrefined maize, soya, ragi (Eleucine coracana, a millet), as well as in Horse gram was ulceroprotective. Amongst these, the lipid from Horse gram was uniquely rich in sterols. Modern processes in the milling of wheat, maize and rice remove these protective lipids [5, 8, 9, 47].

There is increasing evidence that Helicobacter pylori infection may not be a primary cause of duodenal ulceration, but rather lead to non-healing and chronicity [10-12]. In developing countries, where the prevalence of Helicobacter pylori infection is high (80% - 90%), the prevalence of duodenal ulcer is no higher than in countries with a low prevalence of Helicobacter pylori. In China, India and Africa, there are regional differences in duodenal ulcer prevalence despite a uniformly high prevalence of Helicobacter pylori infection. These differences are not accounted for differences in prevalence of virulent strains of Helicobacter pylori [48], but are related to staple diets. The undisputed primary cause of duodenal ulceration remains the gastric secretion of excess hydrochloric acid. In those where hypersecretors acid alone is the probable cause, there is a larger group, whose acid output is less [49] in which some additional factors predispose them to the injurious effect of the acid. The evidence suggests that the presence or absence of protective lipids in the staple diet may be an important factor predisposing to ulceration.

In conclusion, these findings may prove to be of great significance both in reducing the prevalence of duodenal ulceration in areas where it is high, and also in the management of cases of duodenal ulceration, especially those which recur despite eradication of Helicobacter pylori.

There is also another potential use of a combination of both phospholipids and phytosterols, namely to give protection against the ulcerogenic effect of NSAIDs. Earlier experiments with the whole lipid extracted from Horse gram, containing both phospholipid and sterol fractions, showed marked protection against ulceration in the rat aspirin model [13]. Phytosterols have also been reported as giving protection in aspirin models in three other papers [19-21]. As mentioned, Lichtenberger has shown that dipalmitoyl phosphatidyl choline is protective against both aspirin-induced and indomethacin-induced ulceration in rats [34]. It is possible therefore that the combination of phospholipids with phytosterols may be even more potent. A possible source of this could be the oil obtained from fresh wheat bran or wheat germ, pasteurised to prevent lipolysis by the enzymes that are present [9, 47]. This opens up a possible field for further research into the ongoing problem of peptic ulceration and the use of NSAIDs.

## Dedication

This paper is dedicated to the late Dr. Paul Jayaraj, MD, FRCPath, scientist, investigator, and friend who devoted himself to this research throughout the last 20 years before his death.

## References

[1] Tovey F (1979). Peptic ulcer in India and Bangladesh. Gut.

[2] Tovey FI (1992). Duodenal ulcer in China. J Gastroenterol Hepatol.

[3] Wong BC, Ching CK, Lam SK, Li ZL, Chen BW, Li YN, Liu HJ (1998). Differential north to south gastric cancer-duodenal ulcer gradient in China. China Ulcer Study Group. J Gastroenterol Hepatol.

[4] Tovey FI, Tunstall M (1975). Duodenal ulcer in black populations in Africa south of the Sahara. Gut.

[5] Tovey FI, Hobsley M, Segal I, Jayaraj AP (2005). Duodenal ulcer in South Africa: home-pounded versus milled maize. J Gastroenterol Hepatol.

[6] Tovey FI, Jayaraj AP, Lewin MR, Clark CG (1989). Diet: its role in the genesis of peptic ulceration. Dig Dis.

[7] Tovey FI (1994). Diet and duodenal ulcer. J Gastroenterol Hepatol.

[8] Tovey FI (2009). Staple diets and duodenal ulcer prevalence. International Health.

[9] Jayaraj AP, Tovey FI, Clark CG, Hobsley M (2001). Dietary factors in relation to the distribution of duodenal ulcer in India as assessed by studies in rats. J Gastroenterol Hepatol.

[10] Hobsley M, Tovey FI (2001). Helicobacter pylori: the primary cause of duodenal ulceration or a secondary infection?. World J Gastroenterol.

[11] Hobsley M, Tovey FI, Holton J (2006). Precise role of H pylori in duodenal ulceration. World J Gastroenterol.

[12] Hobsley M, Tovey FI, Holton J (2008). Controversies in the Helicobacter pylori/duodenal ulcer story. Trans R Soc Trop Med Hyg.

[13] Jayaraj AP, Tovey FI, Clark CG (1980). Possible dietary protective factors in relation to the distribution of duodenal ulcer in India and Bangladesh. Gut.

[14] Jayaraj AP, Tovey FI, Lewin MR, Clark CG (2000). Duodenal ulcer prevalence: experimental evidence for the possible role of dietary lipids. J Gastroenterol Hepatol.

[15] Paul Jayaraj A, Tovey FI, Hobsley M (2003). Duodenal ulcer prevalence: research into the nature of possible protective dietary lipids. Phytother Res.

[16] Arrieta J, Benitez J, Flores E, Castillo C, Navarrete A (2003). Purification of gastroprotective triterpenoids from the stem bark of Amphipterygium adstringens; role of prostaglandins, sulfhydryls, nitric oxide and capsaicin-sensitive neurons. Planta Med.

[17] Adami E, Marazzi U, Turba C (1962). The anti-ulcer action of some natural and synthetic terpenic compounds. Medicina experimentalis. Med Exp Int J Exp Med.

[18] Xiao M, Yang Z, Jiu M, You J, Xiao R (1992). The antigastroulcerative activity of beta-sitosterol-beta-D-glucoside and its aglycone in rats. Hua Xi Yi Ke Da Xue Xue Bao.

[19] Navarrete A, Trejo-Miranda JL, Reyes-Trejo L (2002). Principles of root bark of Hippocratea excelsa (Hippocrataceae) with gastroprotective activity. J Ethnopharmacol.

[20] Ghosal S, Saini KS (1984). Sitoindosides 1 and 11. Two new anti-ulcerogenicsteryl acyl-glycosides from Musa Paradisiaca. J Chem Research.

[21] Ghosal S (1985). Steryl glycosides and acyl steryl glycosides from Musa paradisiaca. Phytochemistry.

[22] Okuyama E, Yamazaki M (1983). The principles of Tetragonia tetragonoides having an antiulcerogenic activity. I. Isolation and identification of sterylglucoside mixture (compound A). Yakugaku Zasshi.

[23] Lichtenberger LM, Graziani LA, Dial EJ, Butler BD, Hills BA (1983). Role of surface-active phospholipids in gastric cytoprotection. Science.

[24] Dial EJ, Lichtenberger LM (1987). Milk protection against experimental ulcerogenesis in rats. Dig Dis Sci.

[25] Dunjic BS, Axelson J, Ar'Rajab A, Larsson K, Bengmark S (1993). Gastroprotective capability of exogenous phosphatidylcholine in experimentally induced chronic gastric ulcers in rats. Scand J Gastroenterol.

[26] Lugea A, Mourelle, Guarner F, Domingo A, Salas A, Malagelada JR (1993). Phosphatidyl cholines as mediators of adaptive cytoprotection of the rat duodenum. Gastroenterology.

[27] Murty VL, Sarosiek J, Slomiany A, Slomiany BL (1984). Effect of lipids and proteins on the viscosity of gastric mucus glycoprotein. Biochem Biophys Res Commun.

[28] Hills BA, Kirwood CA (1989). Surfactant approach to the gastric mucosal barrier: protection of rats by banana even when acidified. Gastroenterology.

[29] Hills BA (1996). Gastric surfactant and the hydrophobic mucosal barrier. Gut.

[30] Szelenyi I, Engler H (1986). Cytoprotective role of gastric surfactant in the ethanol-produced gastric mucosal injury of the rat. Pharmacology.

[31] Lichtenberger LM, Romero JJ, Kao YC, Dial EJ (1990). Gastric protective activity of mixtures of saturated polar and neutral lipids in rats. Gastroenterology.

[32] Swarm RA, Ashley SW, Soybel DI, Ordway FS, Cheung LY (1987). Protective effect of exogenous phospholipid on aspirin-induced gastric mucosal injury. Am J Surg.

[33] Anand BS, Romero JJ, Sanduja SK, Lichtenberger LM (1999). Phospholipid association reduces the gastric mucosal toxicity of aspirin in human subjects. Am J Gastroenterol.

[34] Lichtenberger LM, Wang ZM, Romero JJ, Ulloa C, Perez JC, Giraud MN, Barreto JC (1995). Non-steroidal anti-inflammatory drugs (NSAIDs) associate with zwitterionic phospholipids: insight into the mechanism and reversal of NSAID-induced gastrointestinal injury. Nat Med.

[35] Lichtenberger LM, Ulloa C, Romero JJ, Vanous AL, Illich PA, Dial EJ (1996). Nonsteroidal anti-inflammatory drug and phospholipid prodrugs: combination therapy with antisecretory agents in rats. Gastroenterology.

[36] Hennessy TM (1992). Effect of membrane plant sterols on excitable cell function. Comp Biochem Physiol.

[37] Haines TH (2001). Do sterols reduce proton and sodium leaks through lipid bilayers?. Prog Lipid Res.

[38] Gupta MB, Nath R, Srivastava N, Shanker K, Kishor K, Bhargava KP (1980). Anti-inflammatory and antipyretic activities of beta-sitosterol. Planta Med.

[39] Bouic PJ, Lamprecht JH (1999). Plant sterols and sterolins: a review of their immune-modulating properties. Altern Med Rev.

[40] Navarro A, De las Heras B, Villar A (2001). Anti-inflammatory and immunomodulating properties of a sterol fraction from Sideritis foetens. Chem Biol Pharm Bull.

[41] Shoji N, Umeyama A, Takei M, Arihara S (1994). Potent inhibitors of histamine release: polyhydroxylated sterols from the Okinawan soft coral Sinularia abrupta. J Pharm Sci.

[42] Takei M, Burgoyne DL, Andersen RJ (1994). Effect of contignasterol on histamine release induced by anti-immunoglobulin E from rat peritoneal mast cells. J Pharm Sci.

[43] Takei M, Umeyama A, Shoji N, Arihara S, Endo K (1995). Mechanism of inhibition of IgE-dependent histamine release from rat mast cells by penasterol and penasterone. J Pharm Sci.

[44] Romero JJ, Lichtenberger LM (1990). Sterol-dependence of gastric protective activity of unsaturated phospholipids. Dig Dis Sci.

[45] Hac-Wydro K, Wydro P, Jagoda A, Kapusta J (2007). The study on the interaction between phytosterols and phospholipids in model membranes. Chem Phys Lipids.

[46] Schuler I, Duportail G, Glasser N, Benveniste P, Hartmann MA (1990). Soybean phosphatidylcholine vesicles containing plant sterols: a fluorescence anisotropy study. Biochim Biophys Acta.

[47] Tovey FI, Hobsley M (2004). Milling of wheat, maize and rice: effects on fibre and lipid content and health. World J Gastroenterol.

[48] Tovey FI, Hobsley M, Holton J (2006). Helicobacter pylori virulence factors in duodenal ulceration: A primary cause or a secondary infection causing chronicity. World J Gastroenterol.

[49] Hobsley M, Whitfield PF (1987). The likelihood of a disease in relation to the magnitude of a risk factor. The example of duodenal ulcer. Theoretical Surgery.

